# WiseEye: Next Generation Expandable and Programmable Camera Trap Platform for Wildlife Research

**DOI:** 10.1371/journal.pone.0169758

**Published:** 2017-01-11

**Authors:** Sajid Nazir, Scott Newey, R. Justin Irvine, Fabio Verdicchio, Paul Davidson, Gorry Fairhurst, René van der Wal

**Affiliations:** 1 School of Engineering, University of Aberdeen, Aberdeen, United Kingdom; 2 The James Hutton Institute, Aberdeen, United Kingdom; 3 Hedmark University College, Campus Evanstad, Evanstad, Norway; 4 School of Biological Sciences, University of Aberdeen, Aberdeen, United Kingdom; West Virginia University, UNITED STATES

## Abstract

The widespread availability of relatively cheap, reliable and easy to use digital camera traps has led to their extensive use for wildlife research, monitoring and public outreach. Users of these units are, however, often frustrated by the limited options for controlling camera functions, the generation of large numbers of images, and the lack of flexibility to suit different research environments and questions. We describe the development of a user-customisable open source camera trap platform named ‘WiseEye’, designed to provide flexible camera trap technology for wildlife researchers. The novel platform is based on a Raspberry Pi single-board computer and compatible peripherals that allow the user to control its functions and performance. We introduce the concept of confirmatory sensing, in which the Passive Infrared triggering is confirmed through other modalities (i.e. radar, pixel change) to reduce the occurrence of false positives images. This concept, together with user-definable metadata, aided identification of spurious images and greatly reduced post-collection processing time. When tested against a commercial camera trap, WiseEye was found to reduce the incidence of false positive images and false negatives across a range of test conditions. WiseEye represents a step-change in camera trap functionality, greatly increasing the value of this technology for wildlife research and conservation management.

## Introduction

Digital technology has a profound effect on environmental monitoring, ecology and conservation [1]. Arguably, this is nowhere better illustrated than in the rapid and widespread uptake of digital camera traps in wildlife research and management [2–4]. Camera traps are remote devices that automatically record images or video (hereafter we use ‘images’ to refer to both still images and video) of animals passing in front of them. The decreasing cost, widespread availability and apparent ease of use offer the potential of collecting large volumes of data that would have been impractical using traditional techniques [2,5]. Ecological application of this technology is diverse [3,6] and includes species inventories [7], assessment of species diversity, community structure and habitat use [8,9], and population estimation [10].

Although camera traps are now widely used in wildlife ecology and management as a research and monitoring tool, the development of most commercially available off-the-shelf devices has been almost entirely been driven by the needs of North American and European recreational hunters [11]. Amid the rapid uptake and widespread use of camera traps, there is growing concern and increasing recognition of their limitations and ability to deliver accurate, robust and ecologically meaningful data [4,6,12–15]. While some of these problems may be associated with the rapid uptake of a new methodology and uncritical use [4,6], other problems are more closely associated with the design and function of the underlying technologies [11].

A survey that sought the views of 154 researchers using camera traps resulted in a comprehensive critique of current design and a long list of camera trap features desired by the camera trap research community [11]. The list included many specific desirable features, such as faster trigger speed (time from first detection to recording of an image), programmable image resolution and frame rate, programmable time-lapse, dual flash systems (infra-red and white flash), multiple and software-controlled sensors, better use of digital communications (e.g. Wi-Fi), and programmable image metadata stored as Exchangeable image file format (EXIF). Moreover, there was an overarching call for a conceptual change in the design of camera traps towards developing a genuinely flexible system that enables users to tailor camera features and settings to a research question and the conditions of use [11]. Combining all of the aforementioned specific features identified by the camera trap research community [4,11,16,17] into one unit is impractical and undesirable, because not all features are required by all users all the time. However, there is an opportunity to exploit the flexibility that digital technology allows, namely to develop an adaptable, user customisable camera trap that can be configured and reconfigured to reflect a user’s changing requirements.

In the light of the above, we set out to design an open source platform that fundamentally progresses camera trap technology by addressing four key constraints: i) lack of user-definable software settings and hardware configurations; ii) false triggering/occurrence of false positive images; iii) resource-demanding cataloguing of captured images to extract meaningful data; and iv) long time-lag between data collection and the results becoming available to inform research and management. We describe each of these constraints in the following paragraphs.

1. Commercially available camera traps use proprietary technology, effectively prohibiting users from modifying a unit. While the wide range of available makes and models gives users a wide choice of features (e.g. sensor sensitivity, time lapse interval, image resolution) to meet different needs, and some manufactures can provide customised units, camera traps are largely closed units once purchased [6,11,18]. By contrast, an open (source) design would allow users to freely customise the software, and offer freedom to modify or extend the hardware. This would greatly improve the utility of camera traps for ecological applications, enabling researchers to tailor functions to specific research needs. It would also provide opportunities for camera traps to simultaneously collect other environmental data cross-referenced to the imagery.
2. Most commercial camera traps use a design with one or more passive infra-red (PIR) sensors to detect animal presence and trigger the camera to record an image. A PIR sensor relies on detecting both a ‘heat signature’–thermal difference between the animal and the ambient background temperature—and movement [5,19]. Detecting animal presence is the critical function of a camera trap. However, a PIR sensor may fail to detect an animal within the detection zone, leading to a false negative (where the true presence of an animal was not detected). A camera may also be triggered to record an image that does not contain an animal, a so-called false positive. False positive images can arise either from environmental conditions (e.g. sunshine that produces sufficient temperature differential in the detection zone to trigger the PIR sensor), or when an animal triggers a sensor but is outside the field of view of the camera. The occurrence and number of false positive images is rarely quantified, but can amount to large numbers of unwanted images [4]. A large number of false positive images are likely to reduce the camera’s effective operational time by filling data storage and depleting batteries. It can also substantially increase effort in post-collection image processing and storage [20–24]. To date approaches to limit the impact of false positive images have focussed on (semi-)automated processing of post collection images [24,25]. These approaches can substantially reduce the number of images that need sorting by a human; they do not, however, reduce the burden of recording, storing and sorting large numbers of non-informative images. This problem could be reduced if camera traps incorporated on-board image processing to identify potential false positive images. Those identified could either be deleted, or flagged as potentially false positives for later assessment.
3. Successfully deployed camera traps can produce large numbers of useful images. The challenge of storing, cataloguing and extracting useable data from these images is a recurring theme in camera trap literature [13,20–23]. Most camera traps record basic metadata (i.e. date and time) with each image, and some also record user-definable metadata (e.g. camera label, geographic coordinates). These metadata are typically limited and often not easily accessible by the user. Image data management and processing could be made more efficient if the camera trap design allows user-definable metadata to be recorded, either as a part of the filename or—preferably—as industry-standard image metadata (e.g. EXIF) that can be accessed using standard image-management software.
4. Camera traps are typically deployed and, after a period of time, revisited to retrieve data and/or perform maintenance. Data and troubleshooting are therefore retrospective and malfunctions can only be identified by physically checking the camera and/or images, often months later, with the risk of serious loss of data collection opportunities [12]. Wireless networking and other digital communication technology have the potential to revolutionise data collection and environmental monitoring [26,27] by allowing remote access to data, status reports and even two-way communication between a PC and camera trap [28]. Some camera trap models now include wireless or cellular communications, but advances in digital communications have not been widely exploited in their design [29].

In this paper we describe the design, implementation and testing of WiseEye, an open source camera trap platform designed to address the four key constraints described above.

## Materials and Methods

### Approach, Design, and Solutions

**Fig 1:**
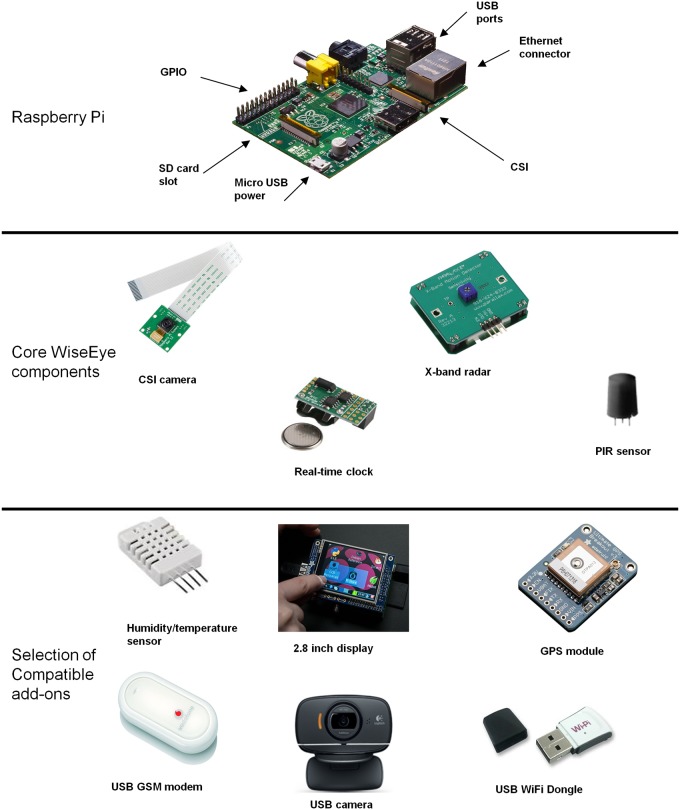
Raspberry Pi with the core components of WiseEye and examples of sensors and peripherals that could be added.

WiseEye is built around a Raspberry Pi 2 model B computer [30] that controls all aspects of the camera trap function. The Raspberry Pi is low cost, small (100 x 60 x 15 mm) and has low power requirements. It is flexible, with a capacity to support readily available peripheral devices and sensors, and there is an extensive user-community support network. The WiseEye software (S1 Appendix) is written in Python [31] and uses a modular architecture that can be readily assembled and integrated to form a working camera trap (Fig 1). This allows the system to be easily modified.

WiseEye operates from a 12 volt battery supply, down-converted to 5 volts to power the Raspberry Pi and peripherals. During development and testing a 50 Ampere-hour sealed lead-acid battery was used, providing continuous field operation for approximately 10 days. For extended deployment, a solar power panel and charge controller can be readily connected (S2 Appendix). While a range of digital cameras could be used, the current design uses a Raspberry Pi infrared camera (NOIR v1) with a frame resolution of 5 mega pixels. Image capture can be motion activated and/or generated from setting up the programmable time-lapse interval. A PIR sensor was selected as the primary sensor to detect the presence of animals in front of the camera and to trigger recording of an image. In addition, we included microwave radar [32] as a confirmatory sensor, operating at 10.525 GHz with an adjustable detection range from 2.4–9 m. The complete unit is housed in an IP-66 weatherproof enclosure.

#### Hardware and software flexibility

**Fig 2:**
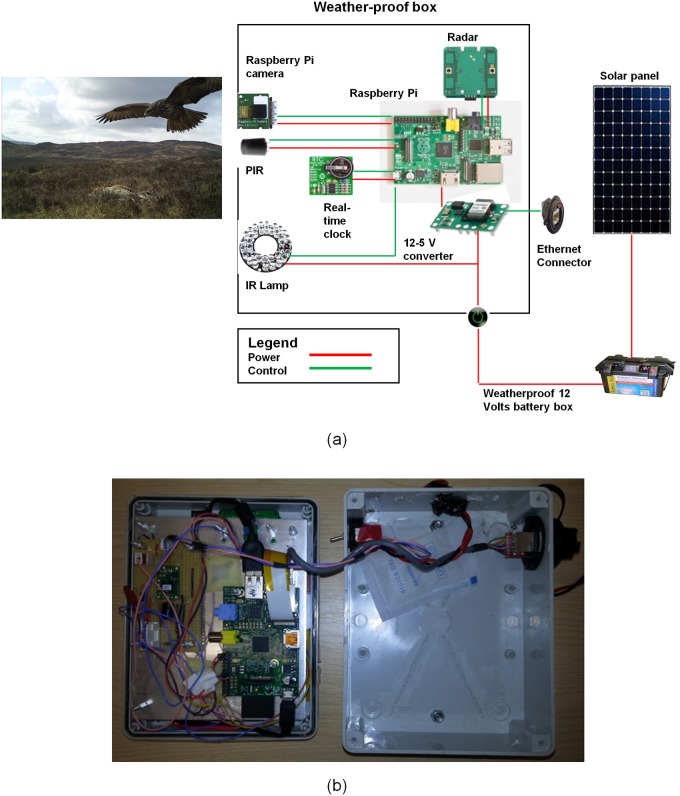
The WiseEye system. (a) Simplified diagram showing inter-connection of components with red lines indicating the power supply and green lines depicting the control/information flow from/to Raspberry Pi. (b) Inside view of WiseEye. The waterproof box has the dimension of 150 x 200 x 80 mm.

The Raspberry Pi model B has a clock speed of 700 MHz and 17 General Purpose Input/Output (GPIO) pins to connect and control sensors and other peripherals (Fig 2). The camera was connected to the Raspberry Pi via a dedicated Camera Serial Interface (CSI). There is a Universal Serial Bus (USB), allowing other USB peripherals such as a 3G dongle or camera to be added, and an Ethernet port. A Real Time Clock (RTC) was added to retain the system time when power is disconnected and to enable the system to be activated or deactivated at pre-programmed times. The Linux-based operating system [33] and the software to control WiseEye, together with captured images and program output, are stored on a memory card in the integral SD card slot. The cost of components is provided in S1 Table.

The WiseEye software automatically starts when the unit is turned on by reading the system settings from a configuration file (a text file specifying variable values for e.g. trigger delay, PIR sensitivity and the time interval between successive images, and other user definable settings). A user can change these settings by editing the file and transferring the updated file to WiseEye before deployment. This eliminates the need to individually set up each camera in the field, eliminating the often fiddly process of navigating on-camera menus to set each parameter individually for each camera (see [4]). This also ensures that settings are not lost or changed to a default setting if power is lost or the system restarts itself.

The user-definable software is a combination of Raspbian [33] and open source products released under a General Public License (GPL). Users can modify and design their own software; this provides an opportunity to release further updates of WiseEye under a GPL. The software uses three self-contained program partitions (classes): i) Sensor (sensor code); ii) ImageProcessor (camera and image processing code); and iii) Controller (program control). This division of functionality across well-partitioned program units makes it straightforward for users to modify the software. All aspects of the hardware and software are under user control, offering considerable scope for user customisation (S1 Appendix). The architecture of the hardware allows WiseEye to be expanded, for example by including a GPS module, environmental sensors, or wireless communication modules (e.g. Wi-Fi, satellite and cellular networking) (Fig 1).

#### Improving detection reliability

A key aim of WiseEye was to improve detection reliability. WiseEye employs two forms of confirmatory sensing to reduce the occurrence of false positive images. The first is a radar sensor [34] to confirm PIR detections. This approach can be implemented so that an image is recorded only when both the PIR and radar detect an animal, but this may delay recording of images. Therefore, in the current implementation the PIR triggers the camera to record an image, and radar activity is logged and cross-referenced to the image data for subsequent analysis. The second form of confirmatory sensing uses the processing capacity of the Raspberry Pi to carry out on-board image processing to identify potential false positive images. The method uses a *Background Subtraction* procedure [35] (as implemented here this process only applies to still images; see S3 Appendix for details) to compare a motion-activated image with a recent time-lapse image to identify whether images are different or not (the time-lapse frequency can be set by the user; here we used a 2 minute interval, and by default the background time-lapse was deleted to save on storage space, but could be kept if desired). Motion-activated images that are similar to the background time-lapse image are likely to be false positives. Images identified as false positives can be deleted or marked for further analysis. The results of comparisons are logged. The user can specify a difference threshold below which images would be classified as false positives. Classifying the captured images immediately following sensor activation uses little power and processing time. Another feature of this method is that the software can restrict the processing to a part of the image area, a Region of Interest. This supports applications that require monitoring within a smaller area rather than the entire camera field of view, such as nest predation studies or monitoring animal activity around burrows. It can also be used to disregard a region of the image from the background subtraction process such as clouds moving in the background or the movement of vegetation.

#### Image management and data extraction

To reduce the time required for cataloguing of the captured images, WiseEye can be configured to include user-specified metadata into the filename and/or image file header. A custom string may be added to the default image filename (timestamp format, S2 Table), for example to identify a study site or to differentiate motion-activated images from time-lapse images. WiseEye also allows users the choice of inserting this (or other) information into the image header file together with data from sensors. The metadata is stored as EXIF tags within a JPEG header file, accessible using standard image management software. This allows for images to be readily catalogued and filtered (by, for example, trigger event, i.e. time-lapse vs. motion activation).

#### Communications and remote access

WiseEye communicates over a cellular network using a USB-connected 3G dongle to report status (e.g. number of triggered events). WiseEye can be enabled to communicate remotely over Wi-Fi, cellular networks or use satellite internet [27] using a USB modem or Ethernet connection. Data and status reports could therefore be retrieved from anywhere on the globe using standard open Internet protocols. Such transmissions can be scheduled automatically at a fixed time, or dynamically based on specified events, for instance when the battery has accumulated a certain charge from the solar panels, or whenever a bird in the monitoring area arrives or leaves its nest. Remote communications can reduce the number of visits needed to field sites, remotely diagnose problems and speed up access to data.

### Methods for Characterising Performance

The ability of WiseEye to reduce false positives and false negatives was assessed in relation to a commercially-available mid-range camera (Bushnell TrophyCam model 119437, listed price approximately £180). This model of camera trap is widely used for both recreation and research [36,37]. Our aim was not to evaluate the Bushnell model but rather to assess WiseEye’s detection characteristics (minimum trigger speed and detection zone), and to test the utility of some of the key design features of WiseEye in a context where it would be typical to use a commercially-available unit.

#### Trigger speed and detection zone

The trigger speed is the elapsed time between the motion sensor activation and capture of an image. This was estimated for both WiseEye and the Bushnell by moving a hand in front of the unit while simultaneously starting a stopwatch placed in front of the camera. The trigger speed was determined from the image captured by the camera trap showing the elapsed time on the stopwatch. This procedure was repeated 15 times to determine an average trigger speed. The detection zone for the PIR and camera field of view was determined in a laboratory by marking distances/angles on the floor and triggering a camera to confirm the zones from the captured imagery.

#### Detection reliability

We assessed the efficacy of the confirmatory radar and background subtraction features of WiseEye to reduce false positives and false negatives relative to the Bushnell camera. Experiments were carried out on the roof of a 3-storey building (School of Engineering, University of Aberdeen). This location offered convenient access to a secure and large (6 m × 8 m) flat study area, regularly visited by herring gulls (*Larus argentatus*) and pigeons (*Columba livea domestica*). The WiseEye unit was powered by a battery connected to a solar panel (S2 Appendix). The cameras were deployed for three days at different times of day. Before each trial, the study area was baited with bread to encourage birds to visit the area.

**Fig 3:**
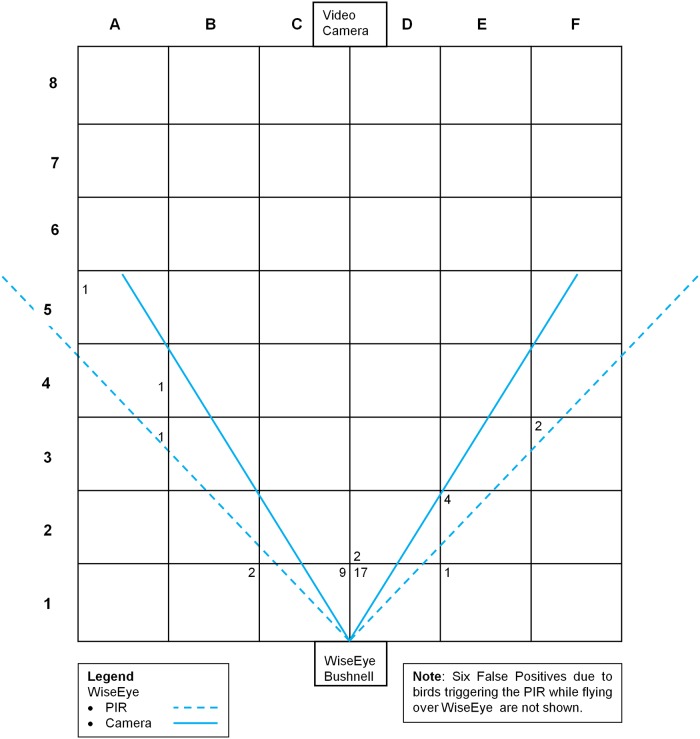
The layout of the roof top study area, showing the positions of the cameras and the detection zone and camera field of view for WiseEye. The numbers on the grid represent the number and locations of False Positive events outside the field of view of the camera.

To assess the rate and occurrence of false positives and false negatives, the study area was simultaneously monitored with a video camera for the period of each trial. To aid interpretation and comparison of the images from the camera traps and the video camera, the study area was marked with a grid of 1 m squares that enclosed the fields of detection/view of both cameras (Fig 3). The WiseEye and the Bushnell cameras were set up side-by-side, 0.8 m above surface level. At the start of each trial the clocks of both cameras and the video camera were synchronized. To prevent multiple images arising from one trigger event, and to give a clear separation of images to allow unambiguous allocation of an image to a detection event, the Bushnell and WiseEye were set up to have a 5 second minimum time interval between two motion-activated images. To assess the effectiveness of the radar to detect the presence of birds, WiseEye was configured to record all radar activity (detections) in the system log file. WiseEye was programmed to also record a time-lapse image every 2 minutes. Background subtraction was used to identify and mark potentially false positive images.

## Results

### Trigger Speed and Detection Zone

The average time to take a picture after a motion event was substantially shorter for WiseEye (0.44 s, standard deviation 0.2 s) than for the Bushnell camera (2.5 s, standard deviation 0.4 s). The WiseEye camera’s field of view was substantially wider than the commercial unit we were using, and the WiseEye PIR detector field of view was wider still (S1 Fig).

### Detection Reliability

**Fig 4:**
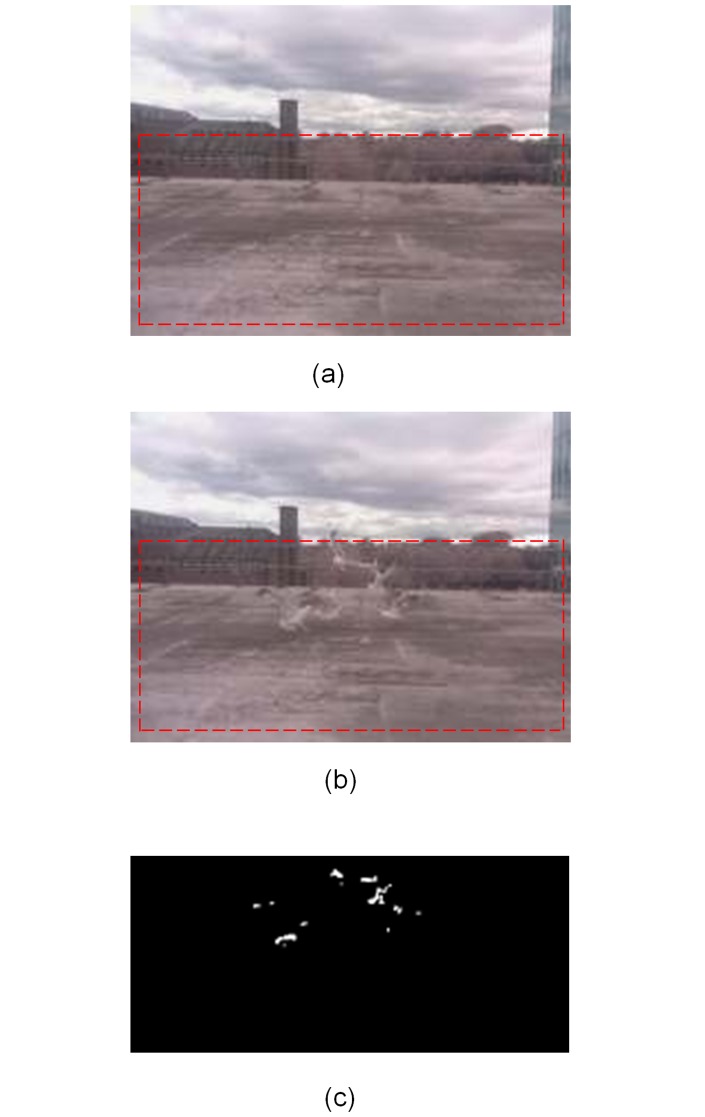
The image processing steps to determine whether an actual target object is present in the field of view by comparing the motion-activated image with a background image. (a) A time-lapse image (with no target) used for subsequent operations as a background image. The Region of Interest (RoI) to be used for image processing is indicated by a red dashed rectangle. (b) motion-activated image showing the objects of interest and RoI. (c) Difference image between the RoI of (a) and (b), showing the birds as clusters of white “difference” pixels.

The trials collected nine and a half hours of video and 232 still images. In line with its greater field of view, WiseEye consistently recorded more (n = 132) true positive images than the Bushnell camera (n = 45) over the course of the three trials (Table 1). Using only PIR detection, WiseEye recorded more false positive images (n = 46) than the Bushnell camera (n = 9). Examination of the video footage showed that all false positives recorded by WiseEye occurred when a bird was present within the PIR detection zone, but outside field of view for the camera (Fig 3). The WiseEye radar sensor only detected 4 of the 132 PIR detections and thus had limited utility (the small body mass of the gulls probably reduced the effectiveness of a radar sensor). By contrast, the WiseEye background subtraction technique successfully identified all 46 false positive images (Table 1, Fig 4), demonstrating that the concept of confirmatory sensing through background subtraction (but not radar) was useful in identifying and potentially eliminating false positives.

**Table 1:** Comparative data for WiseEye and Bushnell camera during the three-day roof top trial. The tabulated numbers refer to the number of images recorded or, in the case of false negatives, the number of potential detections missed.

Image Category	Trigger/Operation	Day					
		1		2		3	
		Bushnell	WiseEye	Bushnell	WiseEye	Bushnell	WiseEye
True Positive	PIR	3	29	4	6	38	97
False Positive	PIR	0	6	1	1	8	39
	After Background Subtraction	N/A	0	N/A	0	N/A	0
False Negative	Video	6	3	4	4	10	8

PIR—Passive Infrared sensor, N/A—Not applicable.

Both cameras suffered from false negative events, though WiseEye failed to detect fewer of those (n = 15) than the Bushnell camera (n = 20) (Table 1). Examination of video footage revealed that many of the birds which WiseEye failed to detect were feeding on the ground and moved too slowly to be detected by the PIR sensor. Additionally, some false negatives occurred during rain, which may have masked the birds’ thermal signature to both cameras.

Thus, WiseEye recorded more false positives than the Bushnell camera trap, but the confirmatory sensing features of WiseEye successfully identified all of these as false positives (and a user could opt to automatically have these deleted). WiseEye was also more effective at detecting animals with a smaller number of false negatives than the Bushnell camera. The lower number of false negatives associated with WiseEye may be due to its wider detection zone and faster shutter speed relative to the Bushnell camera.

## Discussion

The availability of affordable and easy-to-use digital camera traps has allowed the widespread adoption of camera trap technology in wildlife research and management, despite the existence of a number of major constraints [4,6,11,16]. While digital camera traps represent major advances in technology, contemporary camera trap design has generally not exploited the flexibility that digital technology and communications allow and which has been adopted in other areas of environmental monitoring. Below we discuss the innovative features and developments brought by our open source and adaptable platform to camera trap technology and use thereof in the field of wildlife research.

Reliably detecting and recording the presence of animals remains the fundamental purpose of a camera trap, and therefore reducing the rate of false positives is crucial. Two innovations employed by WiseEye offer great potential in this regard. The use of radar to confirm PIR detection as possibly ‘true’ did identify some false positive images, but clearly not many. Although radar itself was not particularly effective here, the principle of ‘confirmatory sensing’ could be applied to other types of sensors. Indeed, the use of on-board, real-time background subtraction to identify differences between time-lapse and motion-activated images effectively eliminated all the false positive images recorded by WiseEye [24]. Alone or together, these two approaches demonstrate the potential of confirmatory sensing to greatly reduce the impact of false positive images and considerably improve survey efficiency and post-collection data storage and extraction. This approach could be combined with more sophisticated routines (e.g. a weighting scheme [38]) to combine input from PIR, radar and image processing to identify a ‘true‘ motion event with even greater accuracy [39].

On-board image processing also paves the way for other novel approaches [40] and applications. For example, the background subtraction procedure could be used to process subsequent time-lapse images, opening up an entirely different way of using camera traps. Previous research demonstrated that variability in detection of different species can be a problem in multi-species surveys, with [13] proposing that time-lapse imagery could be used to overcome this problem. Whilst this recommendation is well founded, implementing time-lapse at a high enough frequency to minimise the risk of missing rarer species inevitably consigns the researcher to excessive post-capture image processing, and still poses the risk of missing rare events. WiseEye’s image processing can address this problem by applying background subtraction to identify images where there is no or a pre-defined level of difference. Real-time processing of (very frequently taken—to not miss rarer species) time-lapse images could be supported by simultaneous use of motion-activation to obtain the benefits of both approaches.

In addition, WiseEye enables a user to specify a Region of Interest, for example to focus on a trail, nest or den entrance. This functionality effectively excludes part of the camera’s field of view, and allows for using the selected area in the background subtraction process. This can be useful when, for example, the image contains moving vegetation in the foreground that might cause false positives and register as a difference if the whole image was evaluated by background subtraction. Thus, specifying a Region of Interest can detect and eliminate false positive imagery.

WiseEye can incorporate metadata (e.g. study site/coordinates, camera label) into the image filename and/or the image header file (as industry-standard EXIF data). This allows user-definable deployment data to be automatically and permanently associated with each and every image at the point of collection, providing functionality repeatedly called for [13,20–23]. Such metadata is easily accessible and extracted using standard desktop packages. This offers substantial improvements in the efficiency of image processing and data extraction, and also enables other sensor data to be easily collated into the same data pool. For example, WiseEye can be equipped with a temperature sensor with the data regularly recorded in the system log file and/or included in the image metadata as an EXIF tag. Data are therefore merged at the point of collection and easily extracted for subsequent analysis.

WiseEye represents a major shift in the ways imagery can be collected for research. With a basic knowledge of Python and electronics users can adapt its hardware and software, both of which are open source. All aspects of WiseEye can be customised to suit specific research needs. The software can be modified so that image or video recording could commence automatically as an animal enters the middle of camera’s field of view, triggered by background subtraction. Hardware can be customised to extend the number or types of sensors. Examples could be: a long-range PIR sensor or sectored PIR sensor to detect movement from a particular direction; a microwave sensor to detect a target in a higher ambient temperature where a PIR sensor would fail; a temperature probe to detect changes in temperature signalling the arrival and departure of a parent bird to and from a nest; a vibration sensor to confirm movement; or a barometric sensor to detect changes in weather. In addition, WiseEye supports flexible communications that lift camera-trap research to a new level by enabling remote data transfer or two-way communication over Wi-Fi, cellular networks or satellite Internet. The built-in Wi-Fi also enables communications with other local sensor-enabled devices, embracing the concept of wireless sensor networks [16,41] and considerably broadening the range of applications of camera traps.

Despite the advantages that WiseEye brings, there are two areas where further development is needed. First, the discrepancy between the detection zone of the PIR and (narrower) field of view of the camera resulted in false positives, because the PIR sensor could detect a slow moving object and trigger image capture before it entered the camera’s field of view. This early triggering contrasts with the longer trigger times associated with many commercial camera traps, for which ‘slow triggering’ can lead to a false positive if an animal moves out of the camera’s field of view before the image is captured. The wider detection zone of the PIR in WiseEye can also be an advantage for a fast moving object, which will be correctly captured in the image if the trigger time is appropriate. Second, the power consumption of WiseEye is substantially greater than that of many commercial camera traps that can operate for weeks or even months on small (e.g. AA cell) dry cell batteries. The current version of WiseEye can be deployed for around 10 days using a 50 Ampere-hour battery weighing approximately 9 kg. It is, however, straightforward to add an external power source such as a solar panel (S2 Appendix). A recent addition controls WiseEye when the camera is active and can be used to optimise power consumption and extend the period of deployment. For example, WiseEye can be programmed to be only active during certain hours (e.g. dawn or dusk) or during certain months, allowing the camera to be deployed months before it is activated to capture imagery. Similarly, there is potential to manage power consumption by programming WiseEye to wait for a time or an external event before activating, such as the air temperature reaching a predefined value.

## Conclusion

WiseEye, an innovative, open-source and adaptable camera trap platform, provides users with the flexibility to customise hardware and software, while allowing ‘plug in’ of a broad range of peripherals and additional sensors. The processing capacity of the Raspberry Pi enables WiseEye to run image analysis in real time combined with the concept of confirmatory sensors. This offers a genuine step change in the way camera traps can be used and represents a paradigm shift in the design of camera traps, opening the way for the use of this tool in a much wider range of contexts.

In the same way that the development of GPS wildlife tracking technology [42], and more recently bio-logging [43], has allowed researchers to ask different questions, the development of camera traps capable of integrating multiple sensor streams and wireless communications in an open source platform has the potential to fast-forward wildlife research and management [15,16,44].

## Supporting Information

S1 Appendix WiseEye User’s Manual. (PDF)

S2 Appendix Solar panel power calculations. (PDF)

S3 Appendix Background Subtraction procedure. (PDF)

S1 Table Cost breakdown by component. (PDF)

S2 Table Format of summary data recorded to CSV file for a motion-activated image. (PDF)

S1 Fig Comparison of the field of view of the PIR sensors and the cameras for the WiseEye and Bushnell. (PDF)
