# Supplementary material for: WiseEye: Next Generation Expandable and Programmable Camera Trap Platform for Wildlife Research
Source: PLoS One. 2017 Jan 11;12(1):e0169758. doi: 10.1371/journal.pone.0169758 (PMC5226779; doi:10.1371/journal.pone.0169758)
Supplement: S1 Appendix — (PDF) [file pone.0169758.s001.pdf]

WiseEye User’s Manual

Sajid Nazir
School of Engineering, University of Aberdeen

1 INTRODUCTION **2**

2 HARDWARE **2**

2.1 PROCESSING UNIT: RASPBERRY PI 2
2.1.1 GPIO *2*
2.1.2 Real Time Clock (RTC) *2*
2.1.3 Camera *2*
2.2 OTHER COMPONENTS 3
2.2.1 Power Supply *3*
2.2.2 Sensors *4*
2.2.2.1 Passive Infrared (PIR) Sensor 4
2.2.2.2 X-band Radar 4
2.2.3 IR LED Panel *4*
2.2.4 Enclosure *4*
2.3 WIRING AND SCHEMATIC DIAGRAMS 5
2.4 COMPONENTS LIST 7

3 SOFTWARE **8**

3.1 SETTING UP A RASPBERRY PI 8
3.1.1 Preparing the SD card for the Raspberry Pi *8*
3.1.2 Configuring the core Pi system on first boot *8*
3.1.3 Enabling Real Time Clock (RTC) *9*
3.1.4 Updating the system *10*
3.1.4.1 Verify your Internet connection using the GUI 10
3.1.4.2 Update the system from the command line 10
3.1.5 Installing Python toolchain *11*
3.1.6 Enabling Services *11*
3.1.7 Setting up a static IP *12*
3.1.8 Setting up a Python script to run on boot as a service *13*
3.2 UTILITY SW TOOLS 13
3.2.1 Download and install Putty and WinSCP *13*
3.2.2 Remote login via Putty *14*
3.2.3 Copying files from the Pi using WinSCP *15*
3.3 PROGRAM FILES IMPLEMENTING WISEEYE 16
3.3.1 WiseEye Source Code *16*
3.3.2 SMS and EXIF code *16*

3.4 CREATE AN IMAGE AND COPY OF SD CARD 17

4 INSTRUCTIONS FOR CONNECTING/DISCONNECTING WISEEYE **17**

4.1.1 Installing and connecting to WiseEye *17*
4.1.2 Disconnecting *18*

5. REFERENCES **19**

6. APPENDIX A-WISEEYE SOURCE CODE **20**

7. APPENDIX B-SMS AND EXIF CODE **28**

1 Introduction

WiseEye is a programmable open source camera trap that makes it possible to tailor the hardware and software to suit a particular application.

The unit, designed to be powered from a 12 V supply, is based on Raspberry Pi. This is a Linux computer with sufficient processing power for image processing and support to interface hardware components like GSM dongle and sensors. The version of WiseEye described here uses two types of motion sensor to detect wildlife activity in front of the camera: a passive infrared (PIR) motion sensor and an X-Band Doppler radar. These detect motion in front of the on-board camera and activate it. The unit can also take pictures during night or in low light using an infra-red (IR) LED panel.

The details of hardware and software components included in the following sections enable the reader to build/modify and operate a WiseEye unit similar to the one described. A list of parts, with manufacturer info, part number and indicative price is given in Section 2.4.

2 Hardware

2.1 Processing unit: Raspberry Pi

The unit is built around a Raspberry Pi 2 Type B [1], shown in **Figure 1**. Later versions (with equal or higher processing capabilities and number of IO pins) can also be used. The Raspberry Pi OS (Raspbian) and working files are stored on a SD card; the same applies to images and data generated during operation. The Pi model 2 Type B requires full-size SD cards. The Raspberry Pi foundation recommends 8GB class 6 cards. Larger capacity (32GB in our experiments) is suggested to be able to store large amount of images.

The details of how to prepare the SD card for Raspberry Pi operation and how to install the required software for WiseEye operation appear later in the document.

2.1.1 GPIO

Raspberry Pi general purpose input output (GPIO) pins are used to interface hardware components. Pins are visible in the top left corner in **Figure 1**; GPIO layout shown in **Figure 2**.

2.1.2 Real Time Clock (RTC)

The RTC is an additional module to the standard Raspberry Pi. This is used to maintain current date/time (only set once) after the unit shut-down (and potentially the battery removal). An example RTC [2] is shown in **Figure 3**. This unit is based on the popular Maxim DS1338 RTC integrated circuit (IC). It is recommended to use an RTC based on either the DS1307 or DS1338 ICs. Please refer to the model specific RTC user manual and instructions for installing and setting up an RTC.

2.1.3 Camera

The Raspberry Pi foundation has released cameras that can be connected to the Raspberry Pi camera serial interface (CSI) connector. The Raspberry Pi camera used in WiseEye is the version 1, 5MP NOIR “no infra-red” [3] camera without the infrared filter to allow use of infra-red flash for low light/night time illumination (**Figure 4**). The other camera for day-light operation [4] can also be used.

2.2 Other components

2.2.1 Power Supply

The power source for the WiseEye unit requires 12V DC. In development and testing we typically used a 6 cell sealed lead acid battery (nominal voltage 12 V). The lead acid 6 cell battery produces most of its power between 12 V and a voltage of approximately 13.6V. It is practically discharged (i.e. unable to supply power) at approximately 9V. The actual fully charged and

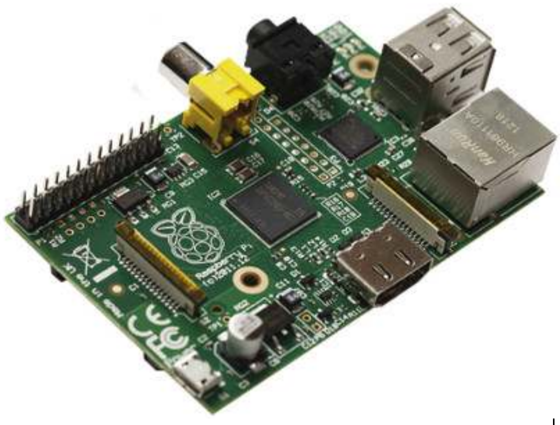

Figure 1: Raspberry Pi 2 Type B

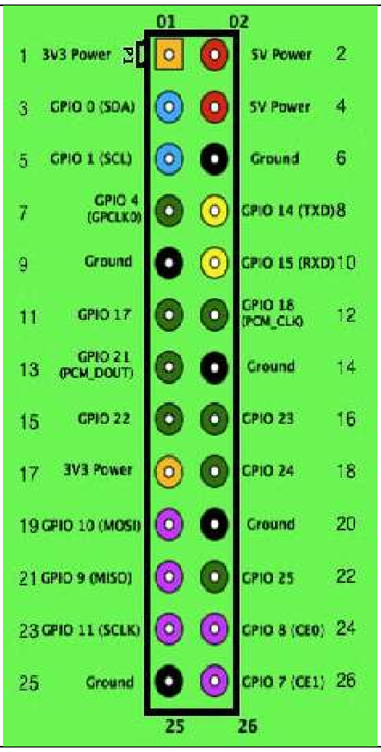

Figure 2: Raspberry Pi GPIO pins scheme

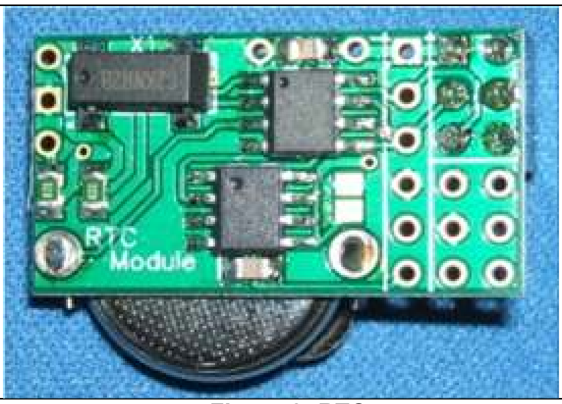

Figure 3: RTC

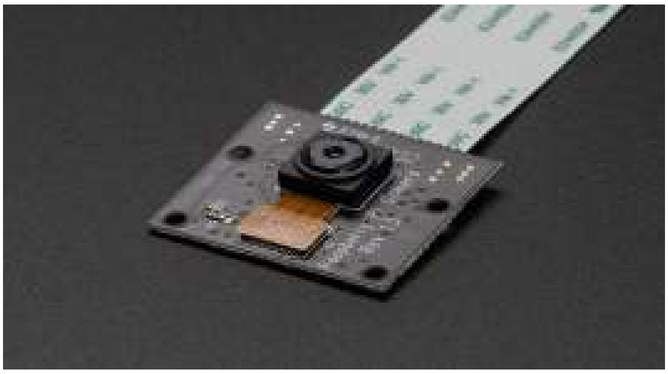

Figure 4: Raspberry Pi Camera

discharged voltages depend upon the battery used and can be found in the manufacturer datasheet. The Raspberry Pi used in WiseEye requires a power supply of approximately 1A at 5V; sensors and cameras fitted to the Raspberry Pi are powered by the Raspberry Pi and are limited to 5V and up to 1A. However, the IR light requires 12V and needs to be connected directly to the battery. To produce the 5V for the Raspberry Pi (and the sensors attached to it) from the 12V source, a DC to DC converter [10] was used. The DC to DC converter supplies a constant 5V to the Raspberry Pi even with decreasing battery voltage. (A cheaper solution would be to use a

7805 voltage regulator, but the voltage delivered to the Raspberry Pi would decline as the battery discharges.)

2.2.2 Sensors

WiseEye uses digital sensors which are described below. The Raspberry Pi lacks an interface for analog sensors; if analog sensors are needed, an ADC board [5] can be used.

2.2.2.1 Passive Infrared (PIR) Sensor

A PIR sensor responds to rapid variations in the thermal radiation received by the sensor. These variations are used to detect an object (warmer than its background) that moves in front of the sensor. The digital PIR [6] requires very little power to operate continuously and can detect changes to about 5 meters from the sensor.

2.2.2.2 X-band Radar

WiseEye uses a 10.525 GHz X-band radar manufactured by Parallax [7], shown in **Figure 5**. The radar requires 8mA at 5V. It has an effective detection range from 2.4m to over 9m. For details refer to the manufactures' user manual [7].

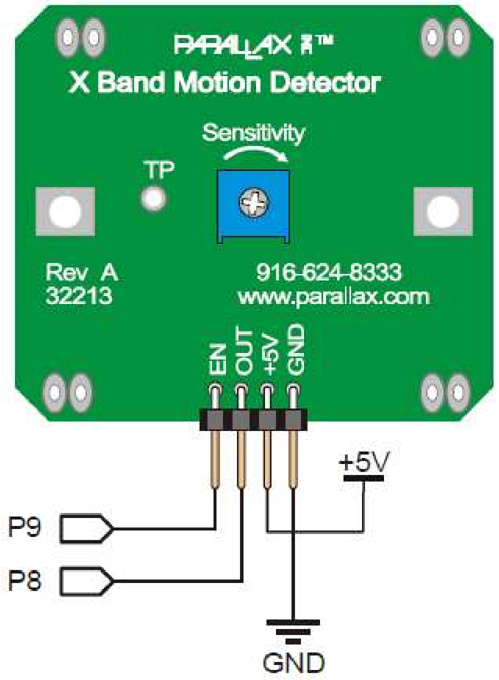

Figure 5: X-band Radar

2.2.3 IR LED Panel

To record images in low light or at night WiseEye requires an (external) infrared light source to. For this purpose WiseEye uses an IR LED panel [8].The panel is powered directly by the 12V battery and it includes a MOSFET Transistor ON/OFF switch that is controlled by the Raspberry Pi.

2.2.4 Enclosure

The platform is housed in a weather-proof casing (IP66). A range of box sizes are available [9]. In addition to water proofing housing we also recommend including silica gel bags inside the box, to absorb any humidity that may otherwise interfere with the electronics.

2.3 Wiring and Schematic Diagrams

WiseEye wiring diagram (**Figure 6**), a schematic showing the overall layout of WiseEye (**Figure 7**), and a picture of the final development version of WiseEye (**Figure 8**) are shown below.

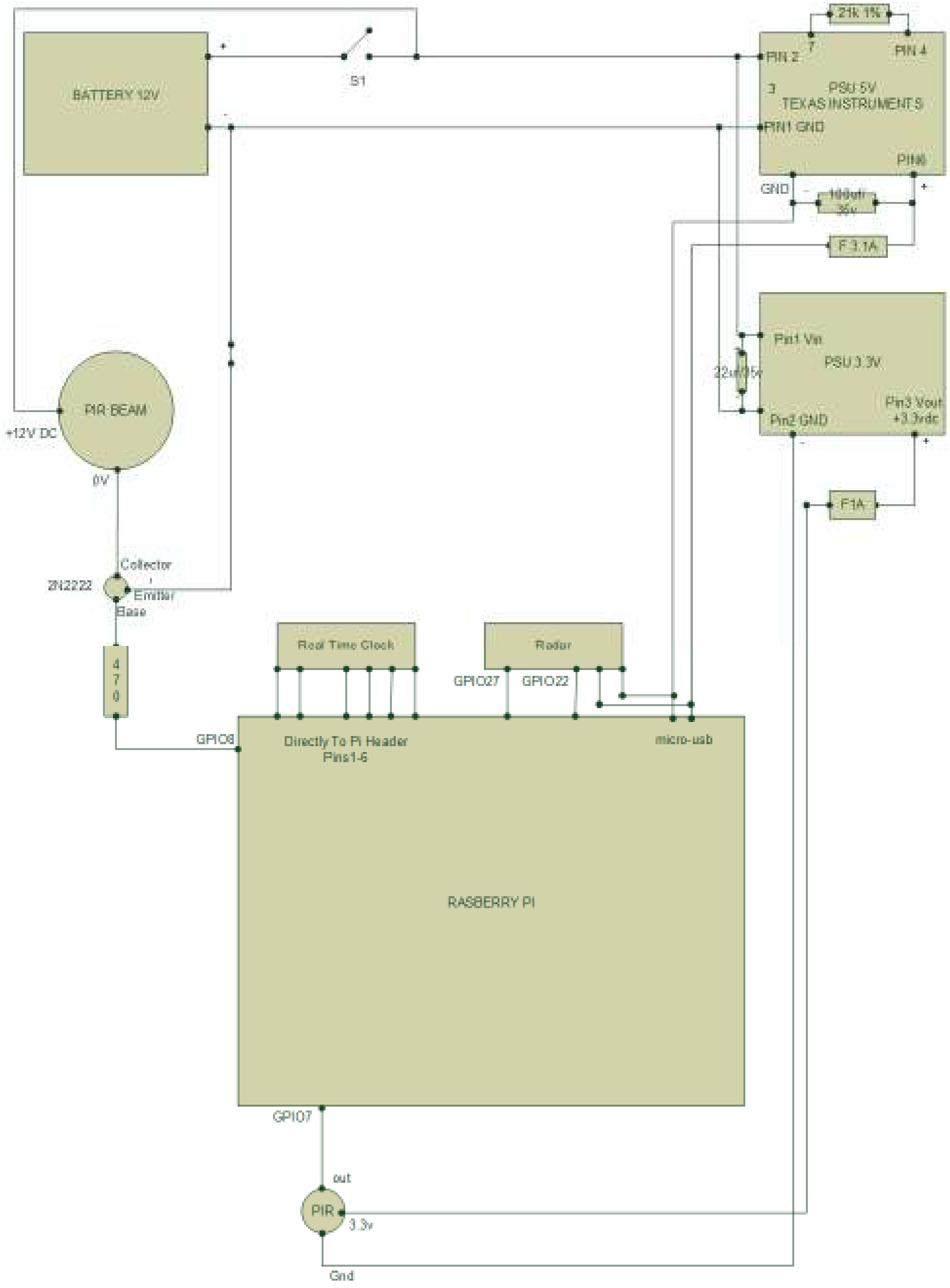

Figure 6: WiseEye wiring diagram.

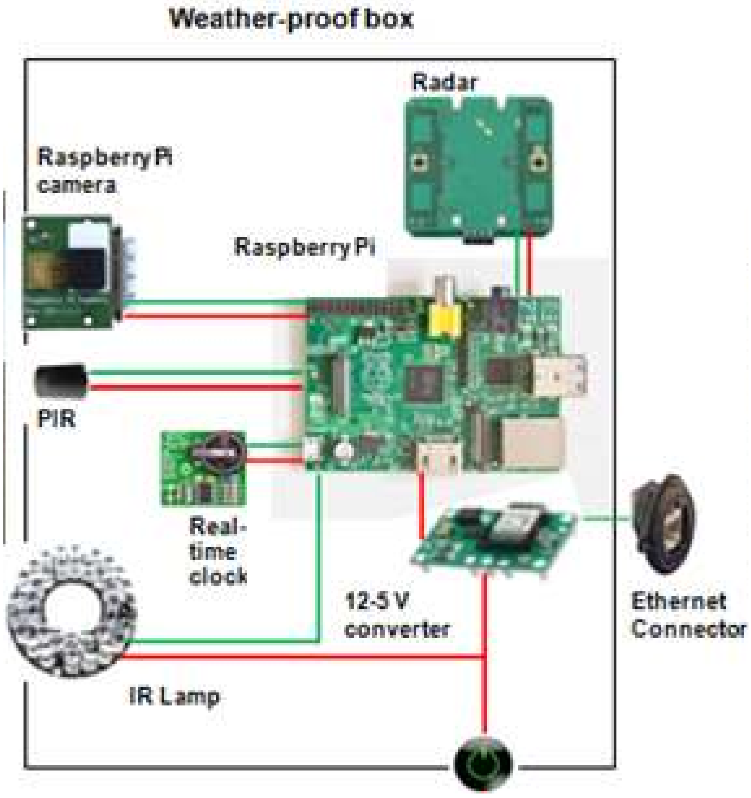

Figure 7: Simplified WiseEye system diagram; inter-connection of components with red lines indicate the power supply and green lines depict control/information flow from/to Raspberry Pi.

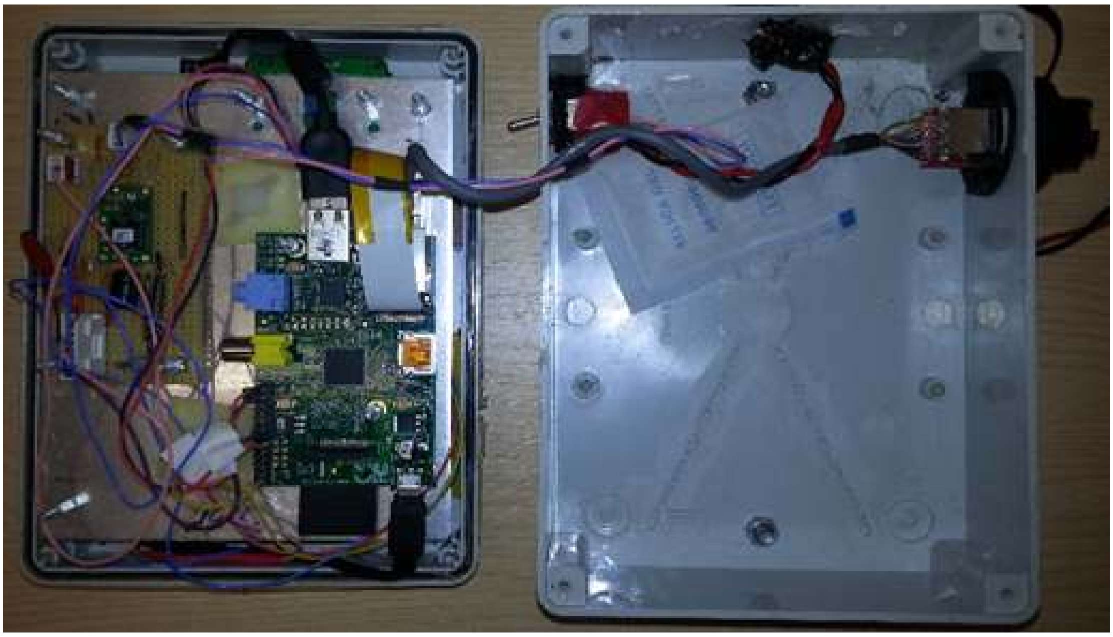

Figure 8: Inside view of WiseEye. The waterproof box has dimensions of 150 x 200 x 80 mm

2.4 Components list

Item Description	Manufacturer/Part number	Retail info	Cost (£)
Raspberry Pi 2, model B	NA	[1]	28.07
Raspberry Pi (NOIR v1) camera	Raspberry PI / RPI-CAMERA	[3]	20.15
Infrared LED panel	TR Computers / NA	[8]	15.99
Real-time clock	4D / RPI-RTC	[2]	10.19
PIR sensor	PANASONIC / EKMC1601112	[6]	6.30
X-band microwave sensor	Parallax / 32213	[7]	40.00
12 to 5V converter	Texas Instruments / PTN78060AAH	[10]	15.44
IP66 enclosure	HAMMOND / 1554C2GY	[9]	25.00

3 Software

3.1 Setting up a Raspberry Pi

The following instructions guide the user to install and configure the OS and required software (SW) tools on a Raspberry Pi. All SW is installed on the SD card. One Raspberry Pi may be used multiple times to perform the processes described below, using a different SD card each time. Alternatively, once one SD card is successfully installed and tested, its content can be duplicated on another card as described in Section 3.4.

It is necessary/best to use a standalone Raspberry Pi to install the OS and SW. The Raspberry Pi used should be connected directly to a USB keyboard, HDMI monitor and internet connection. The Raspberry Pi can be powered using a 5V micro USB supply. The Raspberry Pi powers up automatically when the supply is connected.

Important Note: This document describes the steps required to operate WiseEye on a Raspberry Pi with Raspbian OS (www.raspberrypi.org/downloads/raspbian/). When WiseEye was built, the available Raspbian distribution (version) was called Wheezy. This was later replaced by Jessie. The instructions below rely on core functionalities of (the Linux OS underlying) Raspbian that should work with all distributions. In case of specific problems with a new distribution, please consult the support material from the Raspberry foundation (www.raspberrypi.org) and the internet community at large (via an internet search).

3.1.1 Preparing the SD card for the Raspberry Pi

Aim/objective: This step sets up an SD card so that it can be read by a Raspberry Pi and WiseEye.

Required: SD card, SD card reader, internet connection, and workstation.

These instructions are for the Windows operating system. Different steps are needed for some of these instructions if the SD card is being prepared on a Linux or OS X machine.

Go to www.raspberrypi.org/downloads and download an image file of the latest Raspbian Linux distribution. Then go to http://sourceforge.net/projects/win32diskimager and download and install Win 32 Disk Imager.

Insert the SD card you want to put Raspbian on into an SD card read on the computer that has Win 32 Disk Imager and the Raspbian image. Run Win 32 Disk Imager and select the Raspbian image as "Image File" and the SD card as "Device". You can now write the image to the SD card. Note that this can take up to an hour depending on the SD card and the SD card reader used with the computer.

When the image file has finished been written to the SD card eject the card, insert it in to the stand alone Raspberry Pi and connect a monitor and keyboard for the next stage in the setup process.

3.1.2 Configuring the core Pi system on first boot

Aim: Install and setup Raspbian so that a Raspberry Pi can boot from the SD card.

Required: Raspberry Pi, SD card with Raspbian image, USB keyboard, HDMI monitor.

When the Raspberry Pi boots up the first time you are presented with a setup dialog titled "Raspberry Pi Software Configuration Tool (raspi-config)" with a set of options.

```
1 Expand Filesystem
2 Change User Password
```

```
3 Enable Boot to Desktop/Scratch
4 Internationalisation Options
5 Enable Camera
6 Add to Rastrack
7 Overclock
8 Advanced Options
9 About raspi-config
```

First select option 1 to **Expand Filesystem**. This will allow the Pi to make use of all the storage space available on the SD card.

We also want to enable the camera, so select 5 and then <Enable> to confirm the selection.

Then enter 8 for the **Advanced Options**.

```
A1 Overscan
A2 Hostname
A3 Memory Split
A4 SSH
A5 Device Tree
A6 SPI
A7 I2C
A8 Serial
A9 Audio
A0 Update
```

Here we want to enable **SSH** so select it and choose **<Enable>**. This will enable remote network connections via SSH. Do the same to enable **I2C** to allow connection to the RTC.

Select **<Finish>** on the start screen to complete the configuration. The Raspberry Pi can now be connected to via SSH so the monitor and keyboard are no longer strictly required, but are used below for convenience.

Note: It is also possible to access the Configuration Tool after the first boot, by typing:

```
sudo raspi-config
```

Note: Some instructions/commands require access privileges usually limited to an administrator account (known in the Unix/Linux world as "root"). We can use the 'sudo' command ("Super User DO") in order to perform these commands.

3.1.3 Enabling Real Time Clock (RTC)

Aim: Setup the RTC

Required: Raspberry Pi with Raspbian and RTC connected (as per manufactures instructions), USB keyboard and HDMI monitor.

First, set the system date/time using following command (use the appropriate date/time values):

```
sudo date -s "2 Sep 2014 20:22:00"
```

Check the date/time is correct by typing

```
date
```

Next, set the RTC (hardware clock).

Type the following to open the /etc/rc.local file as root (using sudo). The commands in this file are executed every time the system boots (as if entered by the root user).

```
sudo nano /etc/rc.local
```

The file content will look similar to:

```
#!/bin/sh -e

_IP=$(hostname -I) || true
if [ "$_IP" ]; then
  printf "My IP address is %s\n" "$_IP"
fi

exit 0
```

Here we want to add some commands for enabling the RTC before the exit 0 statement so it looks similar to the following.

```
#!/bin/sh -e

_IP=$(hostname -I) || true
if [ "$_IP" ]; then
  printf "My IP address is %s\n" "$_IP"
fi

modprobe i2c-bcm2708
echo ds1307 0x68 > /sys/class/i2c-adapter/i2c-1/new_device
modprobe rtc-ds1307
hwclock -s

exit 0
```

This will enable the I2C RTC connected to the Pi. Now close and save the file (**CTRL+X** then type 'y' to confirm the changes and hit **Enter** to save the file with the same file name).

Note: The instructions above are for any RTC based on the popular **DS1307** or **DS1338** ICs (as for the RTC used for WiseEye); the RTC instruction manual specifies the model of the IC.

3.1.4 Updating the system

Aim: Update OS and install up to date libraries

Required: Raspberry Pi with Raspbian, internet connection, USB keyboard (optional: USB mouse) and HDMI monitor.

3.1.4.1 Verify your Internet connection using the GUI

First verify that the Raspberry Pi can be connected to the internet via an Ethernet cable. This may require discussing detail of IP allocation with your network administrator.

A way to verify that the Internet connection is working properly is to access a webpage. The easiest way is to use Pi graphical interface (or GUI). Once the Pi has booted, login and then type:

```
startx
```

When the GUI opens, use the mouse/keyboard to select a web browser and open a webpage, e.g. www.google.co.uk and then perform a search. The GUI drop-down menu gives access to a Control Panel to adjust internet settings, if your network administrator requires it.

The GUI drop-down menu also allows opening a terminal, where you can type instructions.

To exit the GUI and return to the console from which you started, press Ctrl+Alt+F1 then Ctrl+C.

3.1.4.2 Update the system from the command line

In order to update the system we use the package manager **apt** that contains a wide SW library:

```
sudo apt-get update
```

This will update the library of software available and prepares to upgrade the whole system:

```
sudo apt-get upgrade
```

This will upgrade all the software on the system. If the system was installed using a recent Raspbian image it will be fairly quick. If an older image was used this may take more than an hour. In either case, do not shut down the system while the upgrade takes place as this can leave the system un-bootable, requiring a full reinstall.

The system is now updated with the most recent software and the upgrade process is finished.

3.1.5 Installing Python toolchain

Aim: Install libraries necessary for WiseEye

Required: Pi with Raspbian, internet connection.

OpenCV is an open source image processing library. It has a couple of dependencies which will be installed before installing OpenCV.

```
sudo apt-get install python-numpy
```

This will install (if not already installed) the NumPy scientific computational library.

Documentation: http://www.numpy.org

```
sudo apt-get install python-scipy
```

This will install (if not already installed) the SciPy package used for numerous scientific applications and simulations.

Documentation: http://www.scipy.org

```
sudo apt-get install libopencv-dev python-opencv
```

This will install OpenCV. The library is large and this installation can take a while.

Documentation: http://docs.opencv.org/modules/refman.html

```
sudo apt-get install python-picamera
```

This will install (if not already installed) the Python interface to the PiCamera module.

Documentation: https://picamera.readthedocs.org/en/release-1.10

```
sudo apt-get install python-serial
```

This will install (if not already installed) the serial library for SMS communication.

```
sudo apt-get install python-pyexiv2
```

This will install (if not already installed) the pyexiv2 library for writing EXIF tags to the JPEG image files.

Documentation: http://tilloy.net/dev/pyexiv2/

3.1.6 Enabling Services

Aim: Setup the RaspberryPi so that the WiseEye software starts automatically on boot.

Requirements: Pi with Raspbian, internet connection.

We want to enable WiseEye to start/restart automatically on power-up. There are two parts to this: one is to install software that enables us to add custom services. Type:

```
sudo apt-get install upstart
```

The second part can be performed later and is discussed in section 3.1.8.

3.1.7 Setting up a static IP

Aim: Setup a static IP for network access to system when WiseEye is running

Required: Pi with Raspbian, monitor and keyboard or network connection.

The configuration for network interfaces can be found in the /etc/network/interfaces file. The file must be opened as root using the sudo command.

```
sudo nano /etc/network/interfaces
```

The file will look similar to this:

```
auto lo
iface lo inet loopback

auto eth0
allow-hotplug eth0
iface eth0 inet manual
```

If the configuration is for general deployment where you want to connect directly to the Pi with an Ethernet cable the configuration needs to look similar to this to set the IP to 192.168.0.0:

```
auto lo
iface lo inet loopback

iface eth0 inet static
address 192.168.0.10
netmask 255.255.255.0
```

If you are using the Raspbian “Jessie” then the configuration is done a bit differently requiring changes to /etc/dhcpcd.conf. The file can be opened as

```
sudo nano /etc/dhcpd.conf
```

At the end of the file of the file add

```
Interface eth0
static ip_address=192.168.0.10/24
netmask 255.255.255.0
```

Note on IP addresses: An IP address is represented with four numbers (each within 0 -255), for example 192.168.0.11. When choosing a static IP it is common practice to select one in the range 192.168.0.0 through 192.168.0.255 as these are reserved to private networks.

To connect the Raspberry Pi to a PC/laptop, this computer must be set with a static IP within the same subnet as the Raspberry Pi: the addresses of the laptop and the Pi should begin with the same three numbers (with Raspberry Pi netmask settings as above). If the Pi address is ‘192.168.0.**10**’, then the laptop IP should be ‘192.168.0.**x**’, with x within 0-255 (but different from 10 as two distinct devices cannot have identical IP addresses); a valid choice for the laptop IP in this case would be ‘192.168.0.**11**’.

The procedure to set a static IP on the laptop depends on its OS and privilege/security settings.

3.1.8 Setting up a Python script to run on boot as a service

Aim: Set up system to run the WiseEye software on boot

Requirements: Pi with Raspbian, internet connection.

The second part to enable WiseEye to start/restart automatically on power-up is to add a configuration file for our service to the /etc/init/ folder. Access this folder, create a file called ‘controller.conf’ and then edit it (using the ‘nano’ editor). To do this, type the following:

```
cd /etc/init/
sudo touch controller.conf
sudo nano controller.conf
```

This will open up the controller.conf which should at this point be a blank file. Use the keyboard to edit the content to be as follows:

```
description "Controller"
start on runlevel [2345]
stop on runlevel [016]
chdir /home/pi/
exec python /home/pi/controller.py >> log.txt 2>&1
respawn
```

Then quit nano (CTRL+X) and save changes to the file (hit Y when prompted to save your file).

In essence, this allows a service, called Controller, to run the WiseEye program soon after the Pi has booted. In more details, the commands above operate as follows. The first line names the auto-running program, or service. The second line specifies that the service starts when the runlevel (OS mode of operation) is 2, 3, 4 or 5 (i.e. the single- and multi-user modes typical of normal operation). The third line specifies that the service stops on runlevel 0, 1 or 6 (essentially, on shut down). The sixth line sets the current working directory to /home/pi/; the following line executes the Python script ‘controller.py’ (with the path /home/pi/controller.py) and redirects the terminal outputs from that script in to log.txt in the working directory. The last line instructs the OS to retry starting the script should this fail to execute (up to a given number of attempts). Any message generated by ‘controller.py’ (including error messages) will be collected and added (appended) to in the log.txt file.

Note: At this point, the user may not have created or copied the file ‘controller.py’ (and other related files) in the home/pi folder. In this case, the service will attempt to run ‘controller.py’ and fail. The error messages will be reported in log.txt. Once the files are copied in the correct directory, as explained in Section 3.3, the service will run as intended (at the following re-boot).

3.2 Utility SW tools

Installing the following software on your laptop allows connecting it to the WiseEye unit in the field. The field operation of WiseEye as described below assumes no keyboard, mouse or display monitor connection to the WiseEye.

3.2.1 Download and install Putty and WinSCP

Aim: Install two pieces of software (Putty and WinSCP) that allow a PC or laptop to network and communicate with a Raspberry Pi / WiseEye unit.

Requirements: PC/laptop, network connection,

Putty is SW that lets you connect to the Raspberry Pi via remote terminal session. Download it (for free) and follow the instructions on http://www.putty.org to install on your pc/laptop.

Similar to the above, WinSCP uses graphical interface ("windows explorer style") for moving files between a remote device and the local PC. Download it (for free) and follow the instructions on https://winscp.net/eng/index.php to install on your pc/laptop.

3.2.2 Remote login via Putty

Aim: Login to the Raspberry Pi from a PC/laptop via Putty

Requirements: PC/laptop with known static IP configured (see Section 3.1.7); Ethernet cable; Pi with SSH enabled (see Section 3.1.2).

Connect an Ethernet cable to WiseEye and connect through Putty (**Figure 9**) using WiseEye IP address (for example 192.168.0.10; recall that your laptop should have a static IP of 192.168.0.x) and log in with user: pi, password: raspberry. The port used for the connection is 22. Successful connection is shown in **Figure 10**.

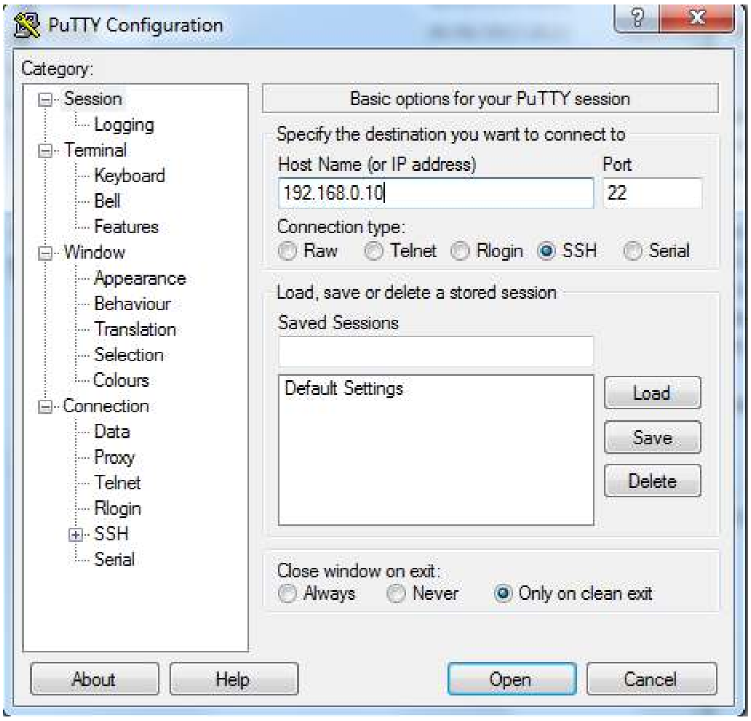

Figure 9: PuTTY interface to connect to Raspberry Pi.

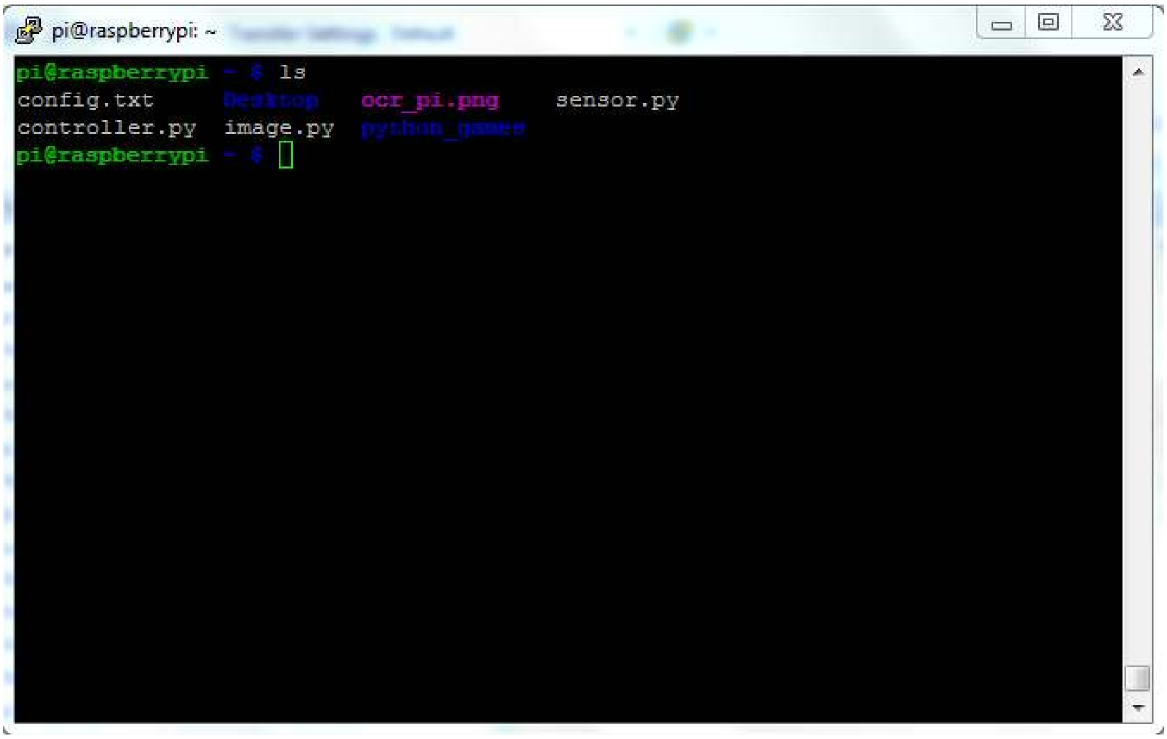

Figure 10: Screenshot of an SSH session.

3.2.3 Copying files from the Pi using WinSCP

Aim: Copying files from Raspberry Pi to PC/laptop using WinSCP

Requirements: PC/laptop with known static IP configured (see Section 3.1.7); Ethernet cable; WinSCP (see Section 3.1.2).

Connect an Ethernet cable to WiseEye and connect through WinSCP (see section 3.2.2; the port used for the connection is 22). You should now be able to connect and transfer files to and from the Pi using a user-friendly graphical interface (**Figure 11**). This provides the following view of the remote (Pi) and local (PC/laptop) devices: one half of the WinSCP window shows the current directory (folder) on the remote device; the other half shows the current directory (folder) on the local device. Choose the intended directory on each device and then drag & drop files between them.

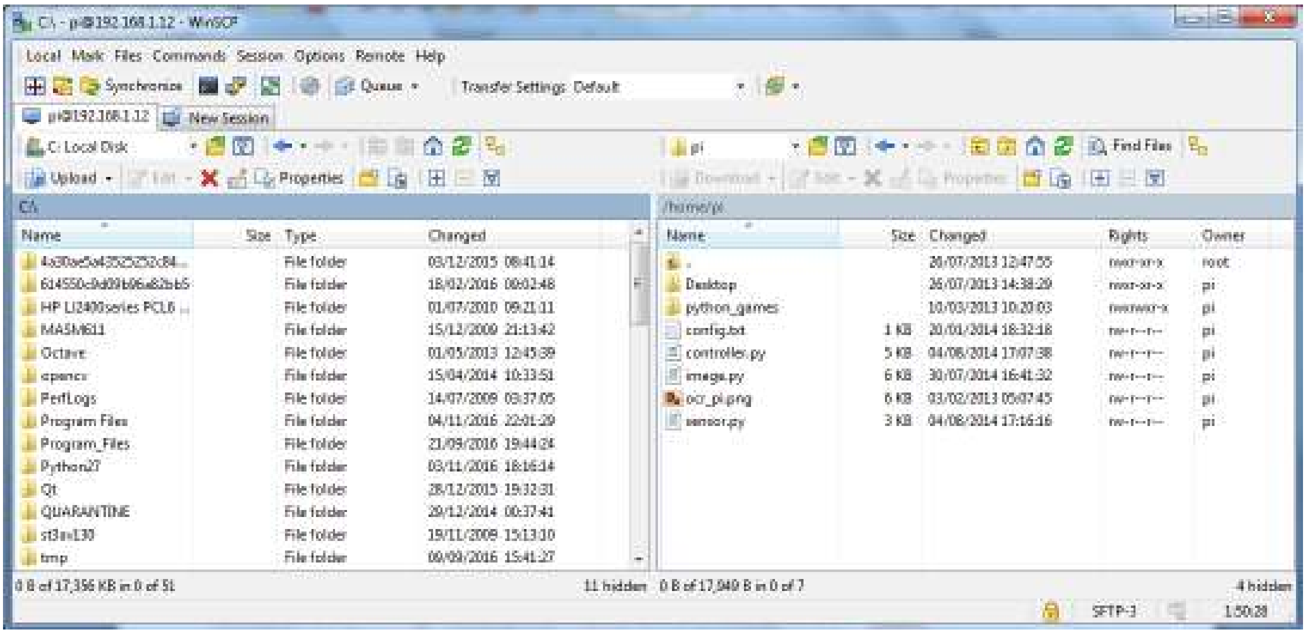

Figure 11: Screenshot of WinSCP interface for file transfer between PC and Raspberry Pi.

Note on visualizing images: Image files (jpeg) may be moved from the Pi to the laptop to be visualized. Once the files are on the laptop, open the relevant directory/folder and access these files with the preferred image viewer. Do not simply double-click on a file inside the WinSCP window to open it. This results in WinSCP opening the file directly: it works with text files (such as log or configuration files), but not with images.

3.3 Program files implementing WiseEye

Aim: Copy the WiseEye files, the software that controls WiseEye (and transforms a Raspberry Pi into a WiseEye camera trap)

The files can be copied, using WinSCP, to the Pi, specifically to folder:

`home/pi`

3.3.1 WiseEye Source Code

The following four files implement WiseEye:

`sensor.py, image.py, controller.py, config.txt.`

The files can be downloaded to a PC/Laptop from https://github.com/sajidnazir/wiseeye/ and are also provided in Appendix A.

When running, WiseEye stores the images taken by the camera in this folder, along with the following files:

`captureData.csv, log.txt`

3.3.2 SMS and EXIF code

The following files implement the SMS communication and EXIF writing to the image file and can be activated through controller.py if required.

`smssend.py, exifwrite.py.`

The files can be downloaded to a PC/Laptop from https://github.com/sajidnazir/wiseeye/ and are also provided in Appendix B.

3.4 Create an image and copy of an SD card

Aim: Allows users to make copies of an SD card that has been fully configured and has the WiseEye files on it to a blank SD card for use in another WiseEye. (Alternatively follow the above instructions again).

Requirements: PC, SD card reader, SD card with installed Linux system to copy from (**source SD card**), SD card of same or greater size to copy to (**destination SD card**).

In order to create an image of an SD card with existing Linux install software such as Win32 Disk Imager available from http://sourceforge.net/projects/win32diskimager is required. Other programs could perform the same job but these instructions are for Win32 Disk Imager.

If required download, install and run Win32 Disk Imager. Insert the **source SD card**, i.e. the card you want to create an image of. In Win32 Disk Imager enter a path and name of the image file you want to create in the “Image File” text box, for example “c:\image.img” which will create image.img in the root of c:. Make sure that the SD card is selected in the “Device” dropdown menu and click “Read” in order to start recording the image. This process can take a while depending on the size of the SD card and the transfer speed. Up to an hour is expected for a 32 GB SD card.

When the transfer is complete eject the SD card that was used to create the image and insert the **destination SD card**, i.e. the one you want to copy the image to. Ensure that the image file you created is entered in the “Image File” text box and that the “Device” dropdown menu is set to the SD card you just inserted. Then click “Write” in order to copy the image file to the SD card. The process will take roughly the same amount of time as the read operation that was previously performed.

When the write process is finished you can eject the SD card. The card should now contain a copy of the original SD card. Repeat the write process for more SD cards as necessary.

4 Instructions for Connecting/Disconnecting WiseEye

The following Python files are the programs in home/pi folder that control WiseEye operation:

sensor.py, image.py, controller.py, config.txt which must not be deleted.

The following files (in addition to images) are generated in home/pi folder:

captureData.csv, log.txt

4.1.1 Installing and connecting to WiseEye

Make sure that the on/off switch on the OFF position.

Connect the battery to WiseEye. Move the switch to the ON position.

Connect an Ethernet cable to WiseEye and connect through Putty using WiseEye IP address (for example 192.168.0.10; recall that your laptop should have a static IP of 192.168.0.x) and log in with user: pi, password: raspberry.

The controller application is programmed to start on connecting power and would be running.

You can verify the status of the controller typing:

```
sudo service controller status
```

The status returned to the prompt should be ‘running’. You can also check the pictures (files) being taken by typing following at command prompt:

```
dir
```

With the program running and pictures being taken orientate the camera to the desired position.

At the command prompt enter:

```
sudo service controller stop
```

This will stop the controller application.

You can delete the images and files (in the current directory) produced by previous executions of the program, using the following command::

```
sudo rm *.jpg
sudo rm *.csv
sudo rm *.log
```

You can restart the controller program by:

```
sudo service controller start
```

4.1.2 Disconnecting

Before switching off or otherwise disconnecting the power, it is recommended WiseEye is shut-down properly, as any other computer.

Connect an Ethernet cable to WiseEye and connect through Putty using WiseEye IP address (for example 192.168.0.10; recall that your laptop should have a static IP of 192.168.0.x) and log in with user: pi, password: raspberry.

At the command prompt enter:

```
sudo service controller stop
```

This will stop the controller application. You can then power-off WiseEye by typing:

```
sudo poweroff
```

After waiting for the Raspberry Pi to switch off (complete system shutdown may take a couple of minutes after the remote terminal connection is lost), turn the on/off switch to the OFF position.

It is now safe to disconnect the battery and remove the system.

5 References

[1] "Raspberry Pi," [Online]. Available: http://www.raspberrypi.org/.

[2] "RTC on Farnell," [Online]. Available: http://cpc.farnell.com/4d/rpi-rtc/raspberry-pi-rtc-module/dp/SC13024.

[3] "RasPiCamNight," [Online]. Available: http://www.amazon.com/Raspberry-Pi-Camera-Filter-Vision/dp/B00G76YEU8.

[4] "RaspPi Camera," [Online]. Available: http://uk.farnell.com/raspberry-pi/rpi-camera-board/raspberry-pi-camera-board-5mp/dp/2302279#.

[5] "ADC for Pi," [Online]. Available: http://www.abelectronics.co.uk/products/3/Raspberry-Pi/39/ADC-DAC-Pi-Raspberry-Pi-ADC-and-DAC-expansion-board.

[6] "PIR on Farnell," [Online]. Available: http://uk.farnell.com/panasonic-electric-works/ekmc1601112/sensor-motion-5m-black/dp/2095729.

[7] "xband radar," [Online]. Available: http://www.parallax.com/product/32213.

[8] "IR panel," [Online]. Available: http://www.amazon.co.uk/illumipi-Infrared-illuminator-Raspberry-Compatible/dp/B00HSSSP2W.

[9] "IP66 box," [Online]. Available: http://uk.farnell.com/hammond/1554c2gy/enclosure-polycarbonate-gry-120x65x40/dp/4080804.

[10] "DC/DC Coverter," [Online]. Available: http://uk.farnell.com/texas-instruments/ptn78060aah/dc-dc-conv-non-iso-pol-1-o-p-15w/dp/2383164?ost=2383164&categoryIdBox=&selectedCategoryId=&iscrfnonsku=false

Appendix A

WiseEye Source Code

a. Sensor.py

```
'''
Copyright 2014 Sajid Nazir <nazirsajid@yahoo.com>

This file is part of WiseEye

WiseEye is free software: you can redistribute it and/or modify
it under the terms of the GNU General Public License as published by
the Free Software Foundation, either version 3 of the License, or
(at your option) any later version.

This program is distributed in the hope that it will be useful,
but WITHOUT ANY WARRANTY; without even the implied warranty of
MERCHANTABILITY or FITNESS FOR A PARTICULAR PURPOSE.  See the
GNU General Public License for more details.

You should have received a copy of the GNU General Public License
along with this program.  If not, see <http://www.gnu.org/licenses/>.
'''

#!/usr/bin/env python2.7
import time
import RPi.GPIO as GPIO

GPIO.setmode(GPIO.BCM)
GPIO.setwarnings(False)


PIR_PIN = 7 #pin for PIR
RDR_SG_PIN = 27 #pin for xband radar signal (input)
RDR_EN_PIN = 22 #pin for enabling radar (output)
IRLED_PIN = 8 #pin for enabling IR LED panel
#TEMP_PIN = 4

def initGPIO():  #for setting pins (as input/output) other than PIR and
Radar
    GPIO.setup(IRLED_PIN, GPIO.OUT, pull_up_down=GPIO.PUD_DOWN) #output
pin for IR LED



def activateIRLED():
    GPIO.output(IRLED_PIN, True) #enable the IRLED for night vision


def deactivateIRLED():
    GPIO.output(IRLED_PIN, False) #disable the IRLED


class Sensor():
    def __init__(self, pin, direction):
        self._sigPin = pin
        self._count = 0
```

```
        GPIO.setup(self._sigPin, GPIO.IN, pull_up_down= direction) #pin
direction as GPIO.PUD_DOWN for PIR and GPIO.PUD_UP for radar
        print 'sensor constructor'
    def getTrigCount(self):
        return self._count #return the number of triggers
    def reset(self):
        self._count = 0
    def enableInterrupt(self, func):
        GPIO.add_event_detect(self._sigPin, GPIO.FALLING) #enable
detection
        GPIO.add_event_callback(self._sigPin, func)
    def disableInterrupt(self, func):
        GPIO.remove_event_detect(self._sigPin)


class PIR (Sensor):
    def __init__(self, pin, direction):     #pin direction as
GPIO.PUD_DOWN)
        Sensor.__init__(self, pin, direction)

    def callbackPIR(self, channel): #count the PIR activations
        self._count  = self._count + 1




class Radar(Sensor):
    def __init__(self, sPin, ePin, direction):     #pin direction as
GPIO.PUD_UP)
        Sensor.__init__(self, sPin, direction)
        self._enPin = ePin
        GPIO.setup(self._enPin, GPIO.OUT, pull_up_down= GPIO.PUD_DOWN)

    def disableRadar(self):
        GPIO.output(self._enPin, False) #disable the radar
    def enableRadar(self):
        GPIO.output(self._enPin, True) #enable the radar
    def callbackRadar(self, channel): #count the Radar activations
        self._count = self._count + 1
```

b. **Image.py**

```
'''
Copyright 2014 Sajid Nazir <nazirsajid@yahoo.com>

This file is part of WiseEye

WiseEye is free software: you can redistribute it and/or modify
it under the terms of the GNU General Public License as published by
the Free Software Foundation, either version 3 of the License, or
(at your option) any later version.

This program is distributed in the hope that it will be useful,
but WITHOUT ANY WARRANTY; without even the implied warranty of
MERCHANTABILITY or FITNESS FOR A PARTICULAR PURPOSE.  See the
GNU General Public License for more details.

You should have received a copy of the GNU General Public License
along with this program.  If not, see <http://www.gnu.org/licenses/>.
'''



from PIL import Image, ImageStat
from scipy import ndimage
import time
import datetime
import math, operator
import os
import subprocess
import cv2
import cv2.cv as cv
import numpy as np
from shutil import copyfile
import picamera

from sensor import *
import threading

class ImageProcessor():
    def __init__(self):
        self._prevImg = None
        self._currImg = None
        self._brightness = 100 #initially consider it daylight
        self._brightness_threshold = 15 #lower values to be considered
dark
        self._time = None #time for the image capture
        self._RPIcam = picamera.PiCamera()
        self._RPIcam.resolution = (640, 480) #set the camera resolution
here
        self._RPIcam.led = False     #switch off camera LED
        print 'image constructor'
    def __del__(self):
        self._RPIcam.close()

    def takePicture(self, type):
        self._time = datetime.datetime.now()
        timeStamp = self._time.strftime('%Y%m%d-%H%M%S')
```

```
        if self._brightness <= self._brightness_threshold:  #its dark
            activateIRLED()#switch-on the IR LEDs
        if type == 'static':
            imgName = timeStamp + '-static.jpg'
            self._RPIcam.capture(imgName)
            self._prevImg = cv2.imread(imgName)
        else:
            imgName = timeStamp + '.jpg'
            self._RPIcam.capture(imgName)
            self._currImg = cv2.imread(imgName)
        deactivateIRLED()#switch off the IR LEDs


    def calculateBrightness(self):
        self._RPIcam.capture("refImg.jpg")
        im = Image.open("refImg.jpg").convert('L')
        stat = ImageStat.Stat(im)
        self._brightness = (int)(((100-0)*(stat.rms[0] - 0))/(255-0)) + 0
#scales a no from min (0 black) to max (255 white) range to 0-100


    def writeImageStats(self):
        #extract ROIs
        ROI_P = self._prevImg[190:460, 20:620]
        roipImgName = self._time.strftime('%Y%m%d-%H%M%S-') +
'roi_static.jpg'
        cv2.imwrite(roipImgName, ROI_P)
        ROI_C = self._currImg[190:460, 20:620]
        roicImgName = self._time.strftime('%Y%m%d-%H%M%S-') +
'roi_motion.jpg'
        cv2.imwrite(roicImgName, ROI_C)
        #compute a difference image on ROIs
        grayp = cv2.cvtColor(ROI_P, cv2.COLOR_BGR2GRAY)  # for
calculating difference image
        grayc  = cv2.cvtColor(ROI_C, cv2.COLOR_BGR2GRAY)   # for
calculating difference image

        diff = cv2.absdiff(grayc, grayp)
        otsu, diff_b = cv2.threshold(diff, 0, 255,
cv2.THRESH_BINARY|cv2.THRESH_OTSU)
        print 'threshold by otsu', otsu
        thresh = round((otsu+self._brightness)/2.0)
        print 'thresh', thresh
        ret, diff_b = cv2.threshold(diff, thresh, 255, cv2.THRESH_BINARY)

        kernel = np.ones((3,3),np.uint8)  #erosion
        diff_erosion = cv2.erode(diff_b,kernel,iterations = 1)
        kernel = np.ones((3,3),np.uint8)  #dilation
        diff_open = cv2.dilate(diff_erosion,kernel,iterations = 1)

        imgName = self._time.strftime('%Y%m%d-%H%M%S-') + 'roi_diff.jpg'
        cv2.imwrite(imgName, diff_open)  #save the eroded/dilated image
as difference


        #calculate the no of blobs in difference image
```

```
        blobs, num_blobs = ndimage.label(diff_open)
        print 'image name' , self._time.strftime('%Y%m%d-%H%M%S')

        contours, hierarchy = cv2.findContours(diff_open,
cv.CV_RETR_LIST, cv.CV_CHAIN_APPROX_NONE) #modifies the image passed as
argument
        max  = 0
        count = 0
        area = 0
        for i in contours:
            area = cv2.contourArea(i)
            if max < area:
                max = area


        #print 'area', area

        if (num_blobs < 30) and ( area > 20):
            verdict = "motion"
        else:
            verdict = "pass"
        return  self._time.strftime('%Y%m%d-%H%M%S'),
self._brightness,thresh, num_blobs, max, verdict
```

c. **Controller.py**

```
'''
Copyright 2014 Sajid Nazir <nazirsajid@yahoo.com>

This file is part of WiseEye

WiseEye is free software: you can redistribute it and/or modify
it under the terms of the GNU General Public License as published by
the Free Software Foundation, either version 3 of the License, or
(at your option) any later version.

This program is distributed in the hope that it will be useful,
but WITHOUT ANY WARRANTY; without even the implied warranty of
MERCHANTABILITY or FITNESS FOR A PARTICULAR PURPOSE.  See the
GNU General Public License for more details.

You should have received a copy of the GNU General Public License
along with this program.  If not, see <http://www.gnu.org/licenses/>.
'''


from sensor import *
from image import *
import os
import csv
import Adafruit_DHT
import sys
```

```
class Controller():
    def __init__(self):        #class initialization
        initGPIO()
        self._param = []       #create an empty list
        self._timeBetweenImages = None #initilaized on reading the config
file
        self._readConfigFile() #populate param from configuration file
                               #assign values from config file
        self._pimg = ImageProcessor()
        self._pir = PIR(PIR_PIN, GPIO.PUD_DOWN )
        self._xbr = Radar(RDR_SG_PIN, RDR_EN_PIN, GPIO.PUD_UP)
        self._myfile = open('captureData.csv','a')
        self._wrtr = csv.writer(self._myfile)
        self._wrtr.writerow(["Serial","Image","Brightness","Threshold",
"PIR Count","Radar Count","No of Blobs","Largest Blob Size","verdict"])
        self._ser = 1

    def __del__(self):             #class destructor
        pass

    def _readConfigFile(self):
        idx = 0
        with open('config.txt') as fp:
            for line in fp:
                line = line.partition(':')[2]
                print line
                self._param.insert(idx, int(line.rstrip()) )
                idx = idx + 1
            #print "index", idx
            self._timeBetweenImages = self._param[0]
            #print 'param', self._param[0]
            #for x  in range(0, idx):
            #    print self._param[x]*2

    def run(self):
        initGPIO()  #set the pins except for radar, and pir
        print 'initializing ...'
        time.sleep(2)
        self._pimg.takePicture('static')
        CAP_PIC = False
        self._xbr.enableRadar()

        while(1):
            self._xbr.enableInterrupt(self._xbr.callbackRadar)
            self._pir.enableInterrupt(self._pir.callbackPIR) #start
listening for activations
            time.sleep(0.05) #sleep for 50 ms
            self._pir.disableInterrupt(self._pir.callbackPIR)

            if ( self._pir.getTrigCount() > 0):   #motion detected by PIR
                start = time.time()
                self._pimg.takePicture('motion')
                p1,p2,p3,p4,p5,p6=self._pimg.writeImageStats()
                self._xbr.disableInterrupt(self._xbr.callbackRadar)  #to
accumulate Radar triggers longer than for PIR
```

```
                self._wrtr.writerow([self._ser, p1,
p2,p3,self._pir.getTrigCount(),self._xbr.getTrigCount(),p4, p5, p6])
                self._myfile.flush()

                self._ser = self._ser + 1

                timeToSleep  =  self._timeBetweenImages  - (time.time()-
start)
                print 'Sleeping for ',timeToSleep
                time.sleep(timeToSleep)

            else:
                lapse = time.strftime('%M')
                if((CAP_PIC == False) and ((int(lapse))%2 == 0) ):
                    CAP_PIC = True
                    #print "time lapse"

                    self._pimg.calculateBrightness() #a n image without
IR LEDs
                    self._pimg.takePicture('static')

                elif (int (lapse) % 2 != 0):
                    CAP_PIC = False
                #self._xbr.disableRadar()   #disable radar for the next
round
                self._xbr.disableInterrupt(self._xbr.callbackRadar)
#execute else:
            self._xbr.reset()   #execute while(1)
            self._pir.reset()



def main():
    con = Controller()
    con.run()

if  __name__ =='__main__':
    main()
```

d. **Config.txt**

time interval between successive images : 5

Appendix B

SMS and EXIF Code

a. **smssend.py**

```
//code adapted from http://stackoverflow.com/questions/2161197/how-to-send-receive-sms-using-at-commands

import serial
import time

class TextMessage:
    def __init__(self, recipient="xxxxxxxxxxx",
message="TextMessage.content not set."):
        self.recipient = recipient
        self.content = message

    def setRecipient(self, number):
        self.recipient = number

    def setContent(self, message):
        self.content = message

    def connectPhone(self):
        self.ser =
serial.Serial(port="/dev/ttyUSB0",baudrate=115200,timeout=0,rtscts=0,xonx
off=0)
        time.sleep(1)

    def sendMessage(self):
        self.ser.write('ATZ\r')
        time.sleep(1)
        self.ser.write('AT+CMGF=1\r')
        time.sleep(1)
        self.ser.write('''AT+CMGS="''' + self.recipient + '''"\r''')
        time.sleep(1)
        self.ser.write(self.content + "\r")
        time.sleep(1)
        self.ser.write(chr(26))
        time.sleep(1)

    def disconnectPhone(self):
        self.ser.close()
```

b. **exifwrite.py**

```
import pyexiv2


def writeEXIF(jpgFile, val):
    metadata = pyexiv2.ImageMetadata(jpgFile)
    metadata.read()

    #print metadata.exif_keys
    exif_uc = 'Exif.Photo.UserComment'
    metadata[exif_uc] = pyexiv2.ExifTag(exif_uc, val)
```

```
    metadata.write()
    return True



#def main():
#    writeEXIF('img2.jpg', 'area-1')


#if __name__ == '__main__':
#    main()
```
