# Supplementary material for: WiseEye: Next Generation Expandable and Programmable Camera Trap Platform for Wildlife Research
Source: PLoS One. 2017 Jan 11;12(1):e0169758. doi: 10.1371/journal.pone.0169758 (PMC5226779; doi:10.1371/journal.pone.0169758)
Supplement: S2 Appendix — (PDF) [file pone.0169758.s002.pdf]

S2 Appendix. Solar panel power calculations.

1. Solar panel kit, 28 Watts (model number: L16BR, www.maplin.co.uk).
2. Specifications:

Power:	28 Watts
Peak Output:	1.70A @ 17.6V
Approximate watt-hours per day:	196
Approximate amp-hours per day:	11.9
Dimensions:	559 x 407 x 25mm
Weight:	2.8kg

3. Battery Input = 12 V x 1.7 A = 20.4 Watts
   for a 12 V battery voltage and 1.7 A peak charge (from the solar panel).

4. The conservative estimated output from solar panel was 6 Watts (instead of 20.4 Watts) based on 10 hours of daylight, sufficient to keep the battery charged to sustain a 2.5 Watts continuous load.
