# Supplementary material for: WiseEye: Next Generation Expandable and Programmable Camera Trap Platform for Wildlife Research
Source: PLoS One. 2017 Jan 11;12(1):e0169758. doi: 10.1371/journal.pone.0169758 (PMC5226779; doi:10.1371/journal.pone.0169758)
Supplement: S3 Appendix — (PDF) [file pone.0169758.s006.pdf]

S3 Appendix. Background Subtraction procedure.

Background Subtraction compares an image with a reference, or background, image I_B to detect potential change. The image detection algorithm first captures a background image using a pre-configured time-lapse at an interval (e.g. every two minutes). When motion is detected by the PIR and/or microwave sensor, the camera triggers capture of a motion-activated image I_M. The image is converted to greyscale and the algorithm subtracts the reference background image I_B from the image I_M to generate a difference image I_D, that identifies the pixels that differ in greyscale intensity between the time-lapse and the motion activated images. The difference (at corresponding pixel locations) between the two images is compared based on a threshold, automatically chosen based on the image content. Let I(x,y) be the intensity of a pixel at location (x,y) in the image then:

$$I_{D(x,y)} = I_{M(x,y)} - I_{B(x,y)}$$

The difference image is then converted to a binary image $I_{DB(x,y)}$ (1 or 0 values, with 1 representing white and 0 black) using the threshold T as:

$I_{DB(x,y)} = 1$ if $I_{D(x,y)} \geq T$
and 0 otherwise.

Areas of the image where pixels have a difference greater than T are represented as a white cluster against a black background. The number of clusters and their size are extracted to provide an indication of (i) the difference between the two images and (ii) the size of the target object that triggered the image. A difference image for two identical images appears as a totally black image. Morphological erosion and dilation operations (http://docs.opencv.org/doc/tutorials/imgproc/erosion_dilatation/erosion_dilatation.html) eliminate small white specks (due to moving vegetation or noise) and extract the size and location of the resulting largest cluster, indicating the position of the target object that likely triggered the camera. The software may be configured to discard (or tag) images with a cluster size below a minimum size, for example; tailored to the size of a particular animal. This can easily be done by ignoring the cluster sizes below a chosen size within a Python program statement. The cluster centre provides an indication of the target position in the image frame.
