# Supplementary material for: WiseEye: Next Generation Expandable and Programmable Camera Trap Platform for Wildlife Research
Source: PLoS One. 2017 Jan 11;12(1):e0169758. doi: 10.1371/journal.pone.0169758 (PMC5226779; doi:10.1371/journal.pone.0169758)
Supplement: S1 Table — (PDF) [file pone.0169758.s003.pdf]

S1 Table. Cost breakdown by component.

Item Description	Cost (£)	
	Full System	Minimum System
Raspberry Pi 2, model B	28.07	28.07
Raspberry Pi (NOIR v1) camera	20.15	20.15
Infrared LED panel	15.99	15.99
Real-time clock	10.19	10.19
PIR sensor	6.30	6.30
X-band microwave sensor	40.00	-
12 to 5V converter	15.44	15.44
IP66 enclosure	25.00	25.00
Weatherproof ethernet connecter	22.00	-
Misc. small components	20.00	20.00
**Sub Total (excluding power)**	**203.14**	**141.14**
Solar panel	119.99	-
12 V deep-cycle rechargeable battery	160.48	50.00
Battery box	64.99	25.00
**Total (including power)**	**548.6**	**216.14**
