# Supplementary material for: WiseEye: Next Generation Expandable and Programmable Camera Trap Platform for Wildlife Research
Source: PLoS One. 2017 Jan 11;12(1):e0169758. doi: 10.1371/journal.pone.0169758 (PMC5226779; doi:10.1371/journal.pone.0169758)
Supplement: S2 Table — (PDF) [file pone.0169758.s004.pdf]

S2 Table. Format of Summary Data recorded to CSV file for a motion-activated image. Line 1 shows the case where PIR detection is not confirmed by radar. Line 2 shows detection confirmation by both PIR and radar. Threshold refers to the value used for background subtraction. 'PIR Count' and 'Radar Count' record the number of times PIR and Radar were triggered for one motion event.

Image Filename	Bright ness	Threshold	PIR Count	Radar Count	No. of Pixel Clusters	Largest Cluster Size
20140124-114409	27	50	1	0	210	75
20140124-114416	27	48	2	5	234	20526
