# Supplementary material for: WiseEye: Next Generation Expandable and Programmable Camera Trap Platform for Wildlife Research
Source: PLoS One. 2017 Jan 11;12(1):e0169758. doi: 10.1371/journal.pone.0169758 (PMC5226779; doi:10.1371/journal.pone.0169758)
Supplement: S1 Fig — (PDF) [file pone.0169758.s005.pdf]

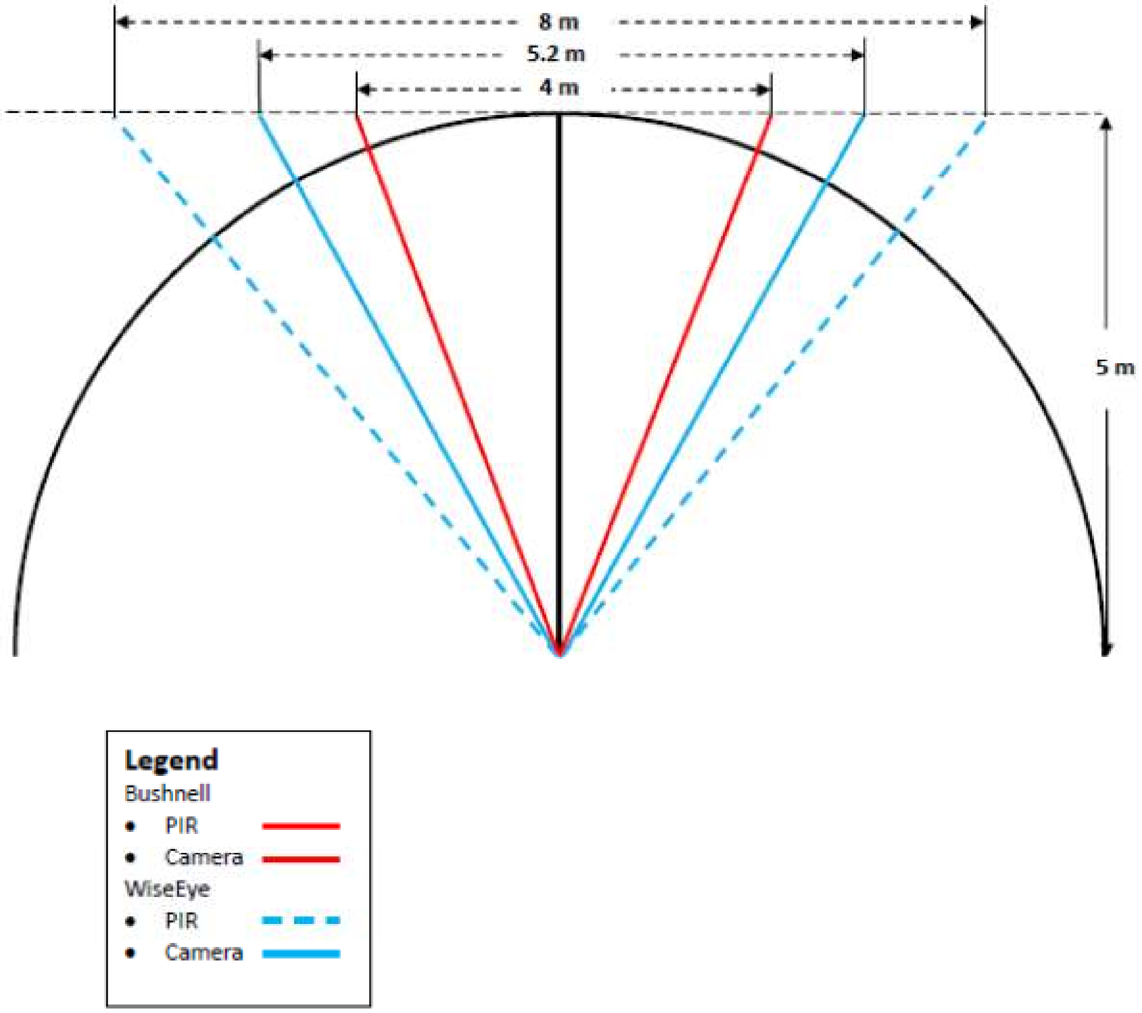

S1 Fig. Comparison of field of view of the PIR sensor and camera for the WiseEye and Bushnell camera traps. The detection zone and camera field of view of the Bushnell are the same. For reference the thick black line shows the 5 m radius.
